# Unexpected co-linearity of Hox gene expression in an aculiferan mollusk

**DOI:** 10.1186/s12862-015-0414-1

**Published:** 2015-08-05

**Authors:** M. Fritsch, T. Wollesen, A.L. de Oliveira, A. Wanninger

**Affiliations:** Department of Integrative Zoology, Faculty of Life Sciences, University of Vienna, Althanstrasse 14, 1090 Vienna, Austria

**Keywords:** Body plan, Development, Evo-devo, Evolution, Mollusca, Novelty, Patterning, Polyplacophora

## Abstract

**Background:**

Mollusca is an extremely diverse animal phylum that includes the aculiferans (worm-like aplacophorans and eight-shelled polyplacophorans) and their sister group, the conchiferans, comprising monoplacophorans, bivalves (clams, mussels), gastropods (snails, slugs), scaphopods (tusk shells) and cephalopods (squids, octopuses). Studies on mollusks have revealed an overall number of 11 Hox genes in seven out of eight molluscan “class”-level taxa, but expression data of key developmental regulators such as homeotic genes are only available for three gastropod and two cephalopod species. These show that Hox genes are involved in the formation of specific features including shell, foot, funnel or tentacles and not in antero-posterior body plan patterning as in most other bilaterian animals. The role of Hox genes in non-conchiferan (i.e., aculiferan) mollusks remains entirely unknown.

**Results:**

Here we present the first data on the expression of seven Hox genes in apolyplacophoran mollusk, *Acanthochitona crinita*. In *A. crinita* the Hox genes *Acr-Hox1-5*, *Hox7* and *Post2* are expressed in a co-linear pattern along the antero-posterior axis, but not in molluscan-specific features such as the shell or the foot. The expression pattern is restricted to the post-trochal region and the transcripts are present in ecto-, endo- and mesodermal cell layers. Contrary to the situation in gastropods and cephalopods, we did neither find Hox gene expression in distinct neural subsets of *A. crinita*, nor in its developing shell plates.

**Conclusions:**

Our analysis and comparison with other lophotrochozoans indicate that the basal role of Hox genes is in antero-posterior axis patterning in mollusks, similar to the vast majority of bilaterian animals, and that this role has been conserved in polyplacophorans, while co-option into patterning of evolutionary novelties emerged either at the base of Conchifera or independently in gastropods and cephalopods. These morphological innovations most likely contributed to the evolutionary success of its representatives, as exemplified by, e.g., the wide ecological range and species richness of gastropods.

## Background

Developmental organization of each particular animal body plan is controlled by a conserved bilaterian-wide cluster of homeotic genes. One of these clusters, the Hox genes, with a conserved homeodomain sequence, possesses a distinctive genomic arrangement and it encodes a set of transcription factors that play a crucial role in organizing the antero-posterior (AP) body axis of many bilaterian animals during development [1–7]. A number of Hox gene expression studies have been published for various bilaterian clades, but most of them are confined to ecdysozoan or deutoterstome representatives (e.g., [8–18]), while data on lophotrochozoans outside the Annelida (e.g., [16–18]) are scarce.

Mollusca is one of the most diverse animal phyla. According to recent phylogenomic analyses [19–22], mollusks contain two monophyletic sister taxa, the primarily univalved Conchifera (Monoplacophora, Scaphopoda, Bivalvia, Gastropoda, Cephalopoda) and the non- or eight-shelled Aculifera (the aplacophoran taxa Neomeniomorpha or Solenogastres and Chaetodermomorpha or Caudofoveata, as well as the shell plate-bearing Polyplacophora). Thereby, recent developmental data have shown that the aculiferans most likely originated from a polyplacophoran-like ancestor, implying that the simple vermiform morphology of the aplacophorans evolved by secondary simplification [23].

Until today, an overall number of 11 Hox genes has been identified in seven out of the eight molluscan “class”-level taxa [24–38], but expression studies on Hox genes are restricted to representatives of two, probably derived, clades of Mollusca that belong to the conchiferans, namely Gastropoda (snails and slugs) and Cephalopoda (squids and octopuses). The existing expression data on Mollusca are limited to larvae of the gastropods *Haliotis asinina*, *H. rufescens* and *Gibbula varia*, and to developing embryos of the cephalopods *Euprymna scolopes* and *Sepia officinalis*. These data show that the Hox genes are involved in the formation of specific morphological features such as the shell, the ganglionic nervous system, the tentacles and the funnel, and that they are - contrary to other bilaterians - expressed in a non-co-linear pattern [24, 26, 27, 33–35, 38]. Furthermore, Hox transcription products could also be identified in sensory organs (e.g., in the apical organ and the statocyst in gastropod larvae) and in the light organ of squid embryos [27, 34]. As opposed to this, in most other bilaterians, Hox genes do show co-linearity and play a crucial role in organizing the antero-posterior body axis [4, 6, 7]. The gastropod and cephalopod expression data suggest that Hox genes may have been co-opted into the formation of novel molluscan morphological features [27, 33–35, 38].

In contrast to gastropods and cephalopods, polyplacophorans possess a number of non-conchiferan characters such as serially arranged shell muscles, dorsal shell plates, as well as a non-ganglionic visceral and pedal nervous system with iterated commissures [23, 39, 40]. In order to assess potential functions of Hox genes in non-conchiferan mollusks, we here present the first data on Hox gene expression in an aculiferan mollusk, the polyplacophoran *Acanthochitona crinita* (Pennant, 1777).

## Results

### Hox gene orthologs and phylogenetic analysis

**Fig. 1:**
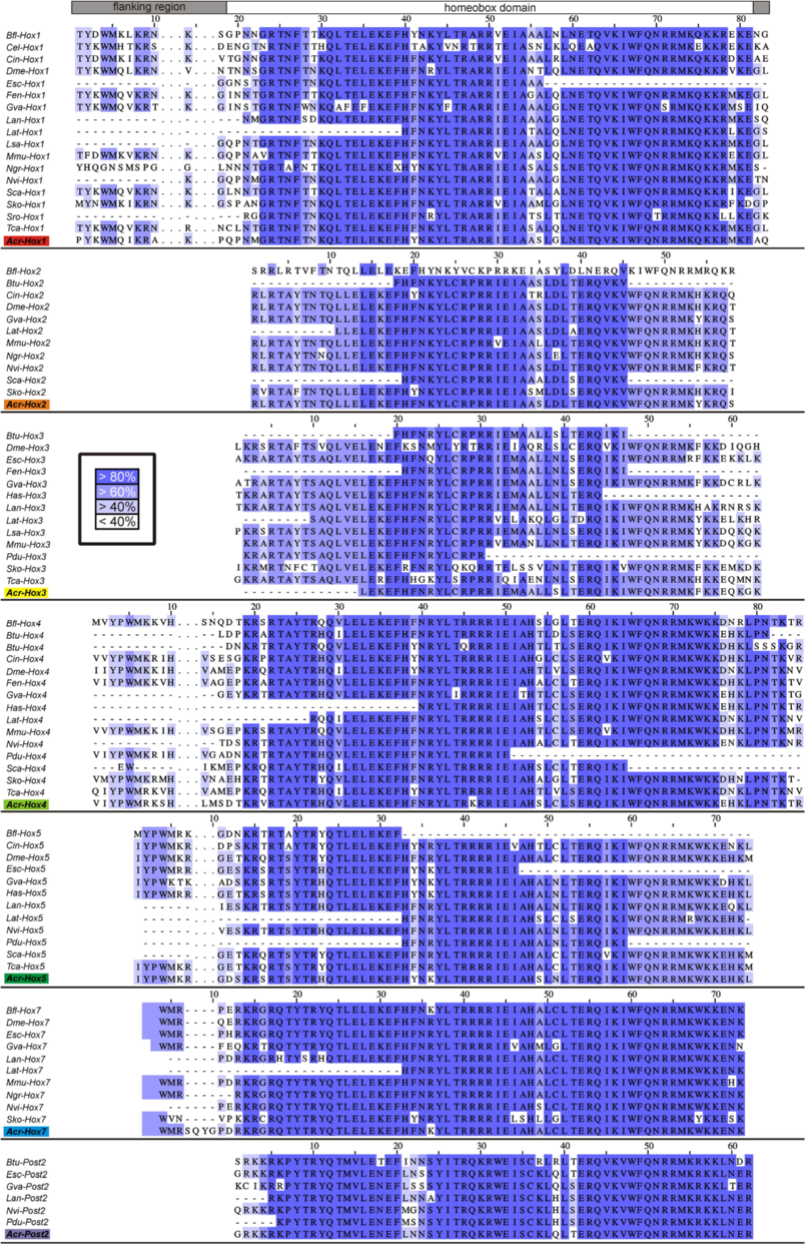
Homeodomain alignment. Sequence alignment containing Hox gene homeo-domains (*Hox1-5*, *Hox7* and *Post2*) and the flanking regions. The residues are bluish colored in each column according to the percentage of identity that agrees with the consensus sequence. Residues with less than 40 % of identity are not colored. Dashes represent missing data

**Fig. 2:**
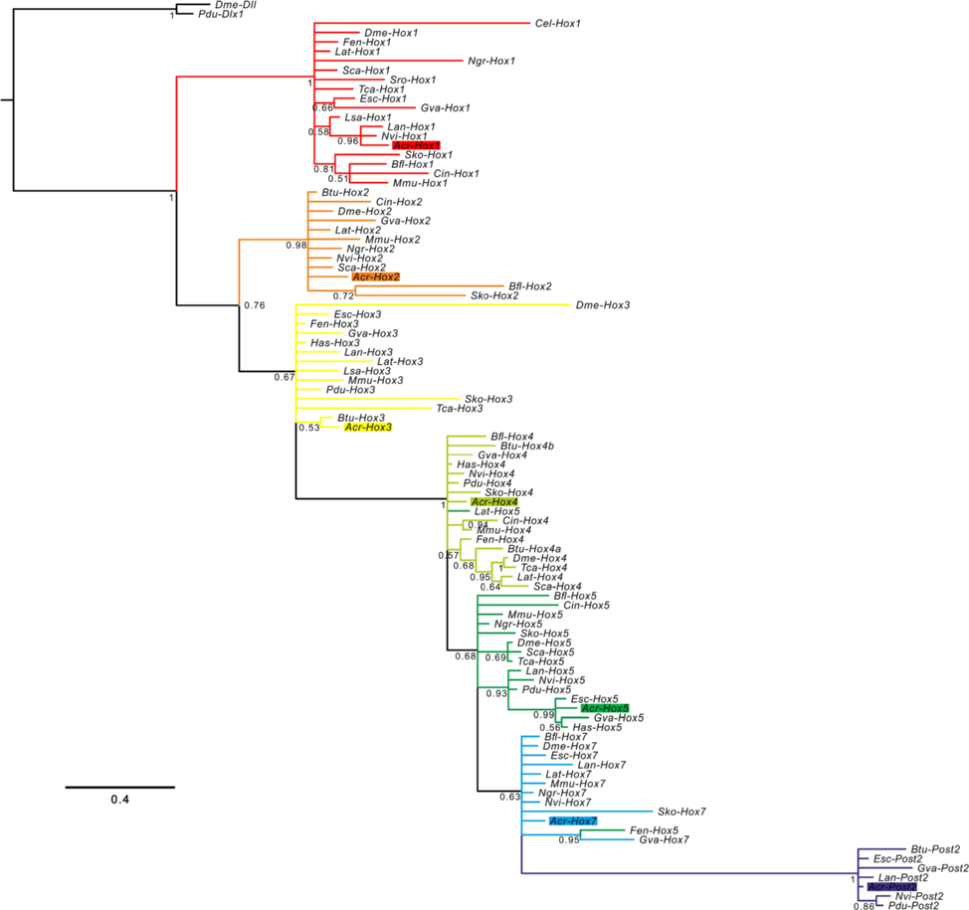
Phylogeny of Hox genes. Phylogenetic reconstruction of Hox genes families from amino-acid sequences present in the homeodomain and the flanking regions. The consensus tree was inferred through Bayesian phylogenetic analysis with MrBayes v3.2.2 discarding 25 % of the samples as burn-in. The branch support values are posterior probability values of Bayesian likelihood. Acr-Hox gene orthologs are highlighted by colored rectangles and the sequences used cluster with other appropriate Hox gene orthologs. The *Hox1*, *Hox2*, *Hox4* and *Post-2* families are strongly supported. Note that branches with posterior probability values less than 50 % are collapsed. The homeobox gene *distalless* was used as outgroup

In the assembled transcriptome of *Acanthochitona crinita* (*Acr*), orthologous Hox gene sequences were identified. The sequences of the genes *Acr-Hox1-5*, *Acr-Hox7* and *Acr-Post2* contain a conserved homeo-domain amino-acid sequence and *Acr-Hox1-2*, *Acr-Hox4-5* and *Acr-Hox7* are flanked by the Y(P)WM-motif at the N-terminal (Fig. 1). All Acr-Hox protein sequences cluster together with their respective bilaterian orthologs, as revealed by the phylogenetic analysis (Fig. 2).

### Larval development of *Acanthochitona crinita*

Early trochophore larvae of *A. crinita* are less than 200 μm long (not shown) and have an apical tuft at the anterior pole. The well-developed prototroch divides the pre-trochal area (episphere) from the post-trochal region (hyposphere). Ventro-medially, posterior to the prototroch, the mouth opening (stomodaeum) is located.

**Fig. 3:**
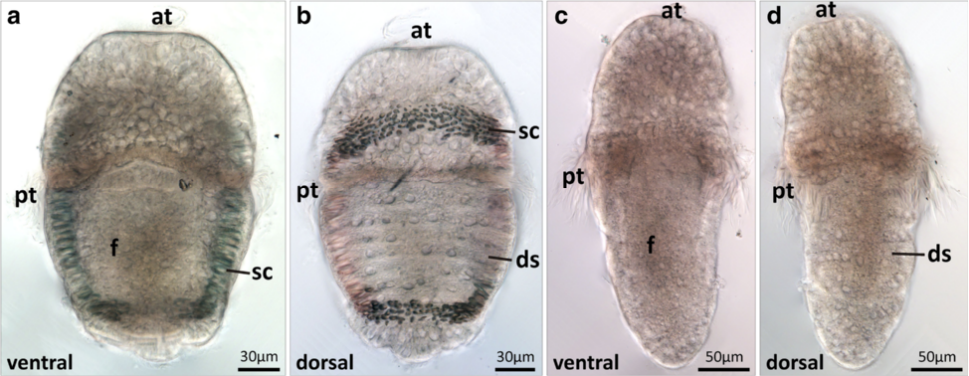
Morphology of *Acanthochitona crinita* trochophore larvae. Anterior faces up. **a**, **b** Mid-trochophore larvae. Episphere and hyposphere are separated from each other by the prototroch. In the dorsal hyposphere region, the *anlagen* of the seven shells are surrounded by spicule-containing cells. **c**, **d** Late trochophore larvae. Seven differentiating dorsal shell plates are present. at apical tuft, ds dorsal shell plates, f foot, pt prototroch, sc spicule-forming cell(s)

Mid-trochophore larvae are oval-shaped and approximately 280 μm in length (Fig. 3a and b). The hyposphere is more elongated, and posterior to the mouth opening the developing *anlage* of the foot extends in the ventral region. Dorso-laterally, on both sides of the hyposphere, a longitudinal row of epidermal and very prominent spicule-containing cells are discernible. Dorsally, an anterior transversal row of spicule-containing cells is present in the episphere and in the posterior region of the hyposphere. In this stage the *anlagen* of the seven dorsal shell plates are already visible in the hyposphere.

Late trochophore larvae are about 360 μm in length and the hyposphere is considerably more elongated than in previous stages (Fig. 3c and d). On the dorsal side of the hyposphere, seven differentiating shell plates are present. At the end of the entirely lecithotrophic, i.e., non-feeding larval development, late trochophore larvae settle and undergo metamorphosis, which is characterized by considerable dorso-ventral flattening of the animals as well as loss of the apical ciliary tuft and the prototroch [41].

### Hox gene expression

#### *Acanthochitona crinita-Hox1*

**Fig. 4:**
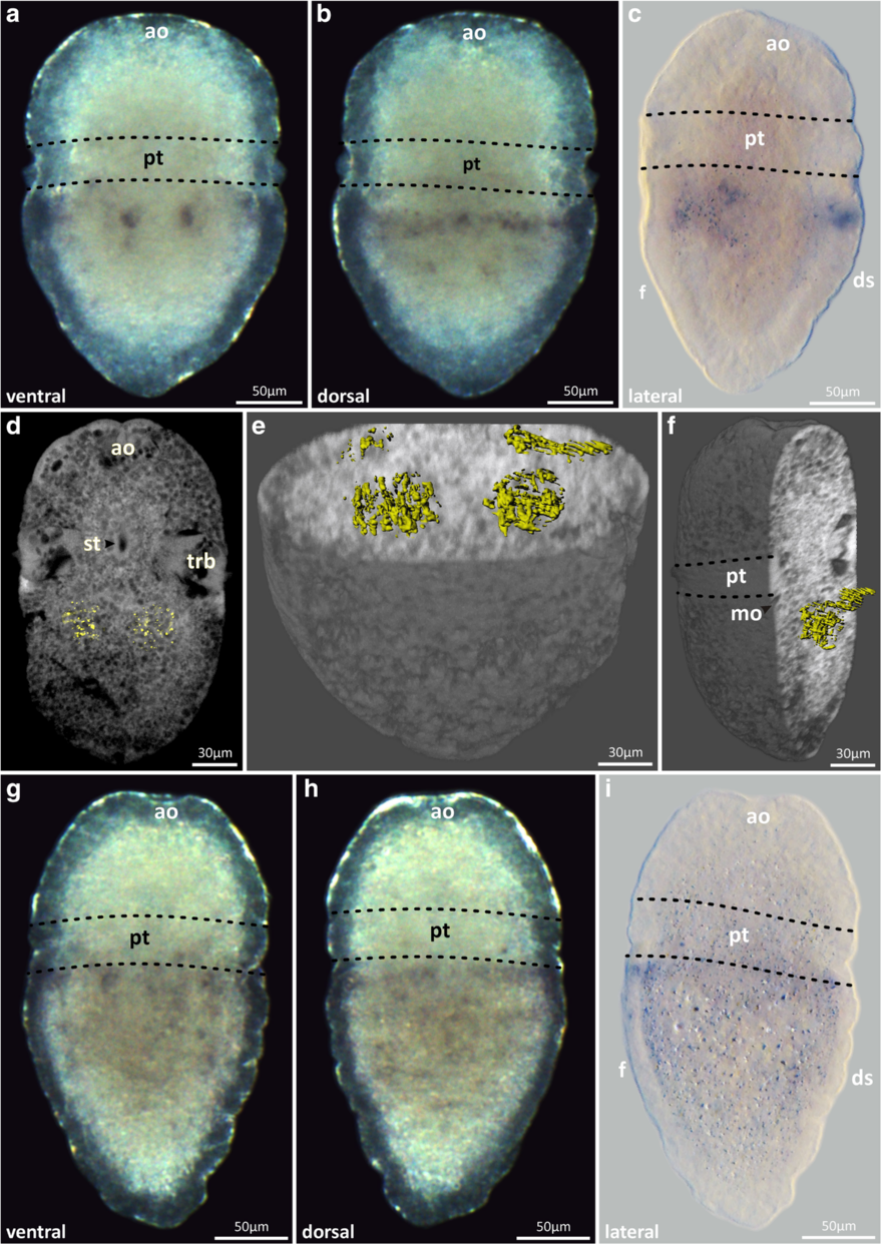
*Acr-Hox1* expression pattern in *Acanthochitona crinita.* Anterior faces up. **a**, **b** Stereomicrographs and **c** light micrograph of mid-trochophore larvae showing the expression pattern of *Acr-Hox1* next to the stomodaeum in the anterior hyposphere. **d**–**f** Autofluorescence (grey) of mid-trochophore larvae and specific transcription product distribution (yellow). **d** Single sagittal section and **e**, **f** ventral and ventro-lateral whole mount clipping plane projection showing the location of the *Acr-Hox1* transcription products in the hyposphere. **g**, **h** Stereomicrographs and **i** light micrograph of the faint *Acr-Hox1* expression pattern in late trochophores. ao apical organ, ds dorsal shell plates, f foot, mo mouth opening, pt prototroch, st stomatodaeum, trb trochoblast(s)

The expression of *Acr*-*Hox1* in early and mid-trochophore larvae is restricted to the anterior region of the hyposphere, postero-lateral to the stomodaeum. Ventrally, the expression pattern is visible in two sub-epidermal cellular spots, lateral to the median body axis (Fig. 4a–f). Dorsally, a slight transversal band of *Acr*-*Hox1* expression is detectable within the epidermal and sub-epidermal cell layer in the anterior region of the hyposphere (Fig. 4c–f). In later stages a weak *Acr*-*Hox1* expression pattern is discernible in the epidermal and sub-epidermal cell layer (Fig. 4g–i).

#### Acr-Hox2

**Fig. 5:**
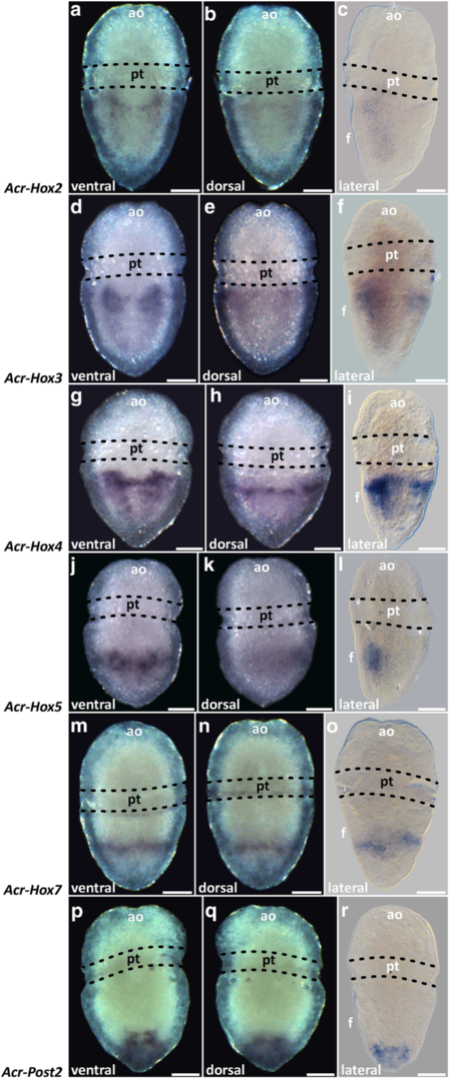
*Acr-Hox2-5*, *Hox7* and *Post2* expression in mid-trochophore larvae of *Acanthochitona crinita.* Anterior faces up. Left and middle column: stereomicrographs. Right column: light micrograph. **a**–**c** *Acr-Hox2* is expressed postero-laterally, next to the stomodaeum, in two ventral, short and parallel longitudinal strands. **d**–**f** Transcripts of *Acr-Hox3* are present in two distinct ventral parallel longitudinal strands. **g**–**i** *Acr-Hox4* is expressed prominently in the hyposphere in two distinct domains which are antero-medially interconnected. **j**–**l** The expression pattern of *Acr-Hox5* is present in two ventral longitudinal strands in the posterior region. **m**–**o** *Acr-Hox7* transcripts are present in a ring-like arrangement in the posterior hyposphere. **p**–**r** The expression pattern of *Acr-Post2* is present at the posterior pole of the larva. Scale: 50 μm. ao apical organ, f foot, pt prototroch

**Fig. 6:**
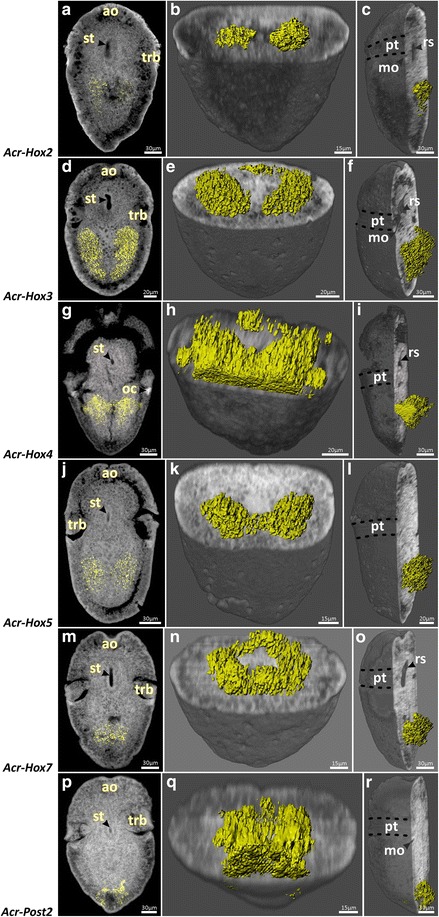
Distribution pattern of the *Acr-Hox2-5*, *Hox7* and *Post2* transcription products in mid-trochophore larvae of *Acanthochitona crinita.* Anterior faces up. Autofluorescence (grey) and specific transcription product distribution (yellow). Left column: single sagittal section. Middle and right column: ventral and ventro-lateral whole mount clipping plane projection. Transcripts of *Acr*-*Hox2* are present sub-epidermally. *Acr*-*Hox3* is ventrally expressed in sub-epidermal cell layers. *Acr*-*Hox4* transcripts are present in the epidermal and sub-epidermal cell layers. Transcripts of *Acr*-*Hox5* are present sub-epidermally. The posterior ring of *Acr*-*Hox7* transcription products is present in sub-epidermal cell layers. *Acr*-*Post2* transcription products accumulate in epidermal and sub-epidermal cell layers. ao apical organ, mo mouth opening, pt prototroch, rs radula sac, st stomatodaeum, trb trochoblast(s)

**Fig. 7:**
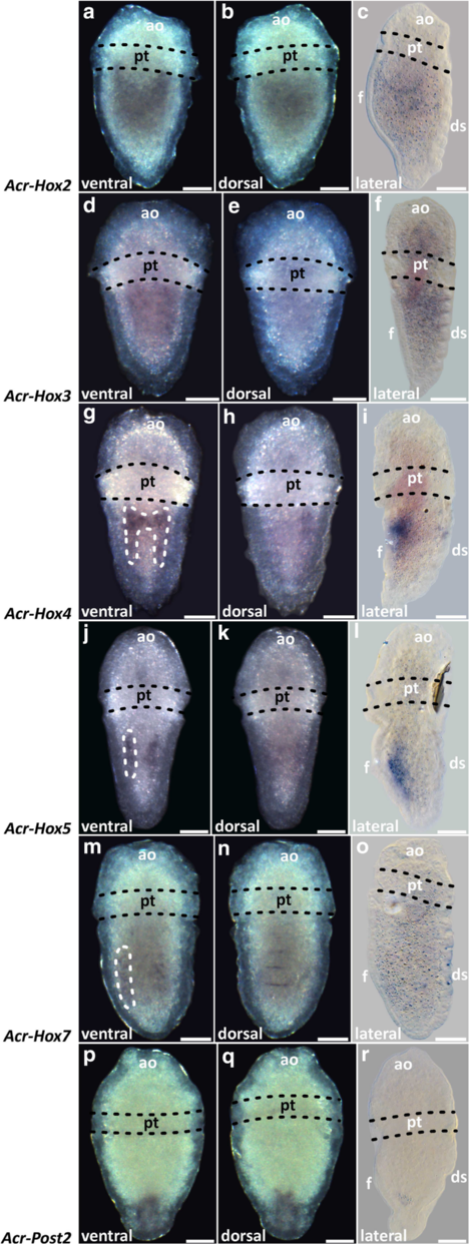
*Acr-Hox2-5*, *Hox7* and *Post2* expression in late trochophore larvae of *Acanthochitona crinita.* Anterior faces up. Left and middle column: stereomicrographs. Right column: light micrograph. **a**–**f** *Acr-Hox2* and *Acr-Hox3* are indistinctly expressed along the antero-posterior larval body axis. **g**–**i** *Acr-Hox4* transcripts are ventrally present, posterior to the stomodaeum and around the lateral edges of the ventral foot. **j**–**l** Two faint *Acr-Hox5* strands are expressed in the mid-region of the hyposphere. **m**–**o** *Acr-Hox7* transcripts are indistinctly present in two postero-lateral expression strands. **p**–**r** *Acr-Post2* expressionis present in the posterior pole of the hyposphere. Scale: 50 μm. ao apical organ, ds dorsal shell plates, f foot, pt prototroch

Transcripts of *Acr*-*Hox2* in early and mid-trochophore larvae are present in two sub-epidermal cell clusters, postero-lateral to the stomodaeum (Figs. 5a–c and 6a–c). The pattern is posteriorly more elongated than in *Acr*-*Hox1* and each expression domain is also located lateral to the median body axis (Fig. 5a). In later stages, an indistinct, faint *Acr*-*Hox2* expression pattern is found sub-epidermally and antero-posteriorly in the hyposphere (Fig. 7a–c).

#### Acr-Hox3

*Acr*-*Hox3* expression pattern in early and mid-trochophore larvae is found in the hyposphere, in two parallel longitudinal sub-epidermal cellular strands (Figs. 5d–f and 6d–f). The pattern is posteriorly much more elongated than that of *Acr*-*Hox2*. Each strand of expression extends laterally to the median body axis. Dorsally, a slight transversal anterior ring of *Acr*-*Hox3* transcripts is present in the hyposphere (Figs. 5e–f and 6e–f). *Acr*-*Hox3* in later stages is only indistinctly expressed in the sub-epidermal layers (Fig. 7d–f).

#### Acr-Hox4

In comparison to the other genes, *Acr*-*Hox4* shows the strongest expression pattern in early and mid-trochophore larvae. *Acr*-*Hox4* transcripts are present in two prominent epidermal and sub-epidermal expression strands in the hyposphere (Figs. 5g–i and 6g–i). Ventro-medially, both elongated strands are interconnected with a slender expression band in the anterior hyposphere region. Laterally, the expression pattern is faint. A slight transversal ring of *Acr*-*Hox4* expression is present antero-dorsally (Figs. 5h–i and 6h–i). Later stages retain a less prominent expression pattern in the ventral hyposphere (Fig. 7g–i).

#### Acr-Hox5

Transcripts of *Acr*-*Hox5* in early and mid-trochophore larvae extend from the central to the posterior region of the hyposphere. *Acr*-*Hox5* is expressed sub-epidermally, in two distinct longitudinal cellular strands (Figs. 5j–l and 6j–l). Antero-medially, both strands of expression are interconnected. In later stages, *Acr*-*Hox5* is less strongly expressed, but still two weak, parallel, sub-epidermal cellular strands are present (Fig. 7j–l).

#### Acr-Hox7

*Acr*-*Hox7* expression in early and mid-trochophore larvae is present in the posterior hyposphere, sub-epidermally in a ring-like pattern (Figs. 5m–o and 6m–o). Expression is ventrally and dorsally more prominent than in the lateral cell layers. *Acr*-*Hox7* is expressed in later stages, indistinctly in the posterior region of the hyposphere (Fig. 7m–o).

#### Acr-Post2

Expression of *Acr*-*Post2* in early and mid-trochophore larvae is in the posterior pole of the hyposphere. *Acr*-*Post2* transcription products accumulate ventrally and dorsally in epidermal and sub-epidermal cell layers (Figs. 5p–r and 6p–r). In later stages, *Acr*-*Post2* is less prominently expressed, but still discernible in the posterior pole of the larva (Fig. 7p–r).

## Discussion

### Hox gene expression in aculiferan and conchiferan mollusks

The existing expression data of three species of gastropods (*Haliotis asinina*, *H. rufescens* and *Gibbula varia* [24, 26, 33–35]) and two cephalopod species (the squid *Euprymna scolopes* and the cuttlefish *Sepia officinalis* [27, 38] have shown that Hox genes are predominantly involved in the formation of the ganglionated central nervous system in these conchiferan mollusks. Other Hox gene transcripts are present in sensory organs such as the apical organ or the statocyst in the gastropod trochophore larvae and in the light organ of *E. scolopes* [27, 34]. Furthermore, Hox gene expression domains include the gastropod shell and the cephalopod brachial crown and funnel.

**Fig. 8:**
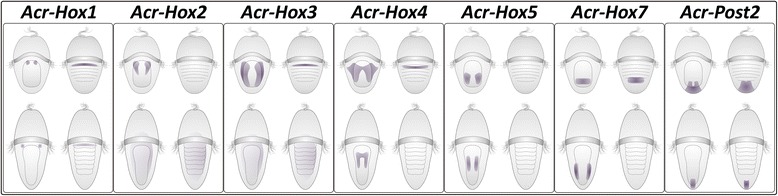
Schematic summary of Hox gene expression in *Acanthochitona crinita*. Each box shows the specific *Acr-Hox1-5*, *Hox7* and *Post2* gene expression patterns in mid (top) and late (bottom) trochophore larvae in ventral (left) and dorsal view (right), respectively

In the polyplacophoran *Acanthochitona crinita*, none of the seven investigated Hox gene orthologs (*Acr-Hox1*-*5*, *Hox7* and *Post2*) show a comparable structural expression pattern during development. By contrast, the Hox genes are expressed in epidermal and sub-epidermal cell layers, in a co-linear manner along the antero-posterior axis in the hyposphere of early and mid-trochophore larvae of *A. crinita* (Figs. 4, 5, 6, 7 and 8).

**Fig. 9:**
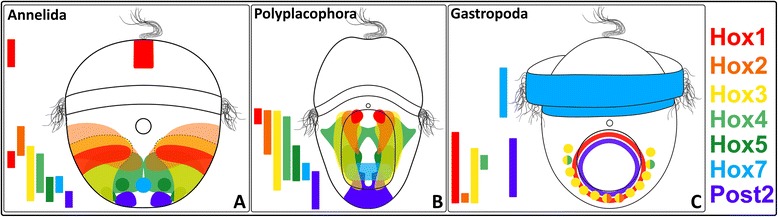
Comparison of Hox gene expression patterns in annelid (**a**), polyplacophoran (**b**) and gastropod (**c**) larvae. Antero-posterior Hox gene expression gradient in the larval body is indicated by colored vertical bars (*Hox1-5*, *Hox7* and *Post2*) on the left side of each trochophore larva. The comparison reveals a relatively similar co-linear Hox gene expression pattern in Annelida and Polyplacophora, whereas in gastropods Hox gene transcripts are expressed non-co-linearly and are often confined to specific morphological features such as the prototroch or the shell field. Annelid data based on *Nereis virens* and *Platynereis dumerilii* [17] and gastropod data based on *Gibbula varia* [33, 34]. Black small circle illustrates the mouth opening, horizontal band the prototroch and ellipse the ventral foot

In early and mid-stage *Acanthochitona crinita* trochophores, *Acr-Hox1* transcripts are found in two ventro-lateral cell clusters, next to the stomodaeum, and dorsally in a slender transversal band. In contrast, in the gastropod *Gibbula varia*, the *Hox1* ortholog (*Gva-Hox1*, Fig. 9c) is expressed in the larval dorso-posterior shell *anlage* [33]. *Acr-Hox2* transcripts are present in two ventral, short and parallel longitudinal strands, postero-lateral to the stomodaeum, and *Acr-Hox3* expression is also present in two distinct ventral parallel longitudinal strands that extend farther in posterior direction than the *Acr-Hox2* expression domain. The *Acr*-*Hox4* ortholog is highly expressed in two domains which are antero-medially interconnected by a slender band. *Gva-Hox2-4* and *Has-Hox2-4* (*Haliotis asinina-Hox2-4*) transcripts are distributed in the ventral ectoderm, postero-laterally around the foot *anlage* [26, 33]. *Acr-Hox5* is expressed in two ventral longitudinal strands in the posterior region of the hyposphere, whereas in gastropod trochophore larvae only a faint endodermal *Gva-Hox5* expression adjacent to the foot rudiment was found [24, 26, 33, 34]. *Acr-Hox7* is expressed in a ring-like pattern in the posterior hyposphere; this pattern differs strongly from that observed in the trochophores of the gastropod *Gibbula varia*, where *Gva-Hox7* is exclusively expressed in the trochoblasts [34]. *Acr-Post2* expression is present in the posterior pole of the hyposphere, while in *Gibbula,* a certain number of cells that surround the posterior foot *anlage* dorsally express *Gva-Post2* [34].

In late *Acanthochitona crinita* trochophore larvae, the expression pattern is faint at best or entirely absent (summarized in Fig. 8), while a distinct distribution of orthologous Hox gene transcripts is present in pre- and post-torsional veliger stages of the gastropods *Gibbula varia* and *Haliotis asinina*. Thereby, *Gva-Hox1, Gva-Post2* and *Has-Hox1* are expressed in the mantle and especially in the shell field [26, 33]. *Gva-* and *Has-Hox2*-*4* transcripts are present in the operculum and in the foot *anlage* [26, 33]. However, the main expression domains of *Gva-Hox2-5* and *Has-Hox2-5*, as well as that of *Haliotis rufescens*-*Hox5* (*Hru*-*Hox5*) and *Gva-Hox7* are in the cerebral, branchial, esophageal, pedal and pleural ganglia [24, 26, 33, 34]. Orthologs of *Hox1*, *3*, *5* and *7* in the embryos of the cephalopod *Euprymna scolopes* (*Esc-Hox1*, *3*, *5* and *7*) are expressed in the developing cerebral, palliovisceral and pedal ganglia, and *Esc-Hox1*, *3* and *Post2* in the stellar ganglion [27]. *Esc-Hox1*, *3*, *5*, *7* and *Post2* are also expressed in cephalopod-specific structures such as the brachial crown and the funnel [27]. As in *E. scolopes*, in embryos of the European cuttlefish *Sepia officinalis* the ortholog of *Sof-Hox3* is also expressed in distinct nervous system and locomotory structures. *Sof-Hox3* transcripts are present in the sub-esophageal masses, brachial, buccal and stellar ganglia and in parts of the funnel [38].

In comparison to the Hox gene expression data on gastropods and cephalopods, the polyplacophoran *A. crinita* Hox gene transcripts are not restricted to particular subsets of the developing nervous system (although we do not rule out the presence of Hox transcripts during neurogenesis). This may be due to the medullary cord-like character of the polyplacophoran nervous system which, in contrast to the gastropods and cephalopods, lacks distinct ganglia [41–43]. Data on polyplacophoran neurogenesis have shown that in early and mid-trochophore larvae of *Ischnochiton hakodadensis* and *Mopalia muscosa* the primary pedal neurons (derivatives of the ectoderm) develop and extend posteriorly [42, 43]. Interestingly, in the post-trochal region in early and mid-stage *A. crinita* trochophore larvae, the transcripts of the *Acr*-*Hox1-5* genes are present in two parallel antero-posterior medial expression domains, which overlap with the region of the developing neurons of the future paired pedal nerve cords. In contrast, in the lateral post-trochal areas of the developing visceral nerve cords, no overlapping Acr-Hox gene expression pattern is present. However, in *A. crinita*, the overall expression pattern is not restricted to, e.g., the developing pedal nerve cords. Instead, the Acr-Hox gene transcripts are also found in more lateral and more central sub-epidermal tissues which clearly do not contribute to neural tissues (Figs. 4–6).

Developmental data on the muscular system have shown that in early and mid-trochophore larvae of *Mopalia muscosa* and *Leptochiton asellus* several muscle fibers start to develop in the post-trochal region (the future dorso-ventral muscles [23, 41]). The Hox gene transcripts in early and mid-stage *A. crinita* trochophore larvae are expressed in a region comparable to that where these (mesoderm-derived) muscles form. However, the Acr*-*Hox gene expression pattern is not restricted to individual muscular structures.

Henry and colleagues [44] labeled derivatives of the endoderm in the polyplacophoran species *Chaetopleura apiculata* via injected fluorescent markers in cell lineage experiments. Thereby, it was found that in early and mid-trochophore larvae of *C. apiculata*, third-quartet macromeres give rise to the developing body wall and the (endoderm-derived) gut in the post-trochal region. In early and mid-stage *A. crinita* trochophore larvae, the transcripts of anterior Hox genes (such as *Acr-Hox1-4*) are present post-trochally around the forming stomodaeum and adjacent to the central area of the forming digestive tract (as part of the endoderm).

Indeed, in the polyplacophoran *Acanthochitona crinita*, the Hox gene expression patterns are not restricted to distinct morphological structures (e.g., ganglia, shell plates, etc.). However, comparable developmental polyplacophoran data and also the Hox gene expression patterns indicate, that the Acr-Hox gene transcripts are seemingly present in tissues derived from all three germ layers (ectoderm, endoderm and mesoderm). These expression patterns differ significantly from the Hox gene expression pattern in gastropods and cephalopods (see above), indicating that Hox genes are involved in different, taxon-specific developmental processes in gastropods and cephalopods, but not in polyplacophorans. In contrast, the polyplacophoran transcripts *Acr-Hox1*-*5*, *Hox7* and *Acr-Post2* are distributed in a specific spatial antero-posterior pattern. Anterior Hox genes (e.g., *Acr*-*Hox1*-*2*) are only expressed in the anterior hyposphere, next to the stomodaeum, and posterior Hox gene transcripts (such as *Acr*-*Post2*) are only present in the posterior hyposphere. This antero-posterior mode of expression resembles that of the co-linear Hox gene expression in the vast majority of bilaterian animals, including Acoelomorpha, Annelida, Ecdysozoa and Deuterostomia (e.g., [14, 16, 17, 26, 34, 45–54]) and thus appears to have been conserved in polyplacophoran mollusks.

In this study, the expression of seven out of the putatively 11 molluscan Hox genes was investigated in the polyplacophoran *Acanthochitona crinita*. Preliminary data on the yet missing genes *Acr-Lox5*, *Lox4* and *Lox2* (*Acr-Post1* could not be identified) are in line with the antero-posterior Hox gene expression pattern described herein.

### Antero-posterior Hox gene expression in Polyplacophora and Annelida

Comparing our results with those of Annelida, a taxon probably closely related to Mollusca (e.g., [19, 36, 55–57]), important similarities in the spatial Hox gene expression patterns along the antero-posterior axis become obvious (Fig. 9a–b). Early trochophore larvae of the annelids *Nereis virens* and *Platynereis dumerilii* show a strikingly similar co-linear Hox gene expression pattern to that of *Acanthochitona* in the ventral hyposphere. In *A. crinita*, the *Acr*-*Hox1* expression is located in the anterior hyposphere; orthologs of *N. virens-Hox1* (*Nvi-Hox1*) and *P. dumerilii-Hox1* (*Pdu-Hox1*) are similarly expressed in the anterior hyposphere, the future first parapodial segment [17]. The *Acr*-*Hox2* expression postero-lateral to the stomodaeum is similar to the stomodeal *Pdu-Hox2* expression [17]. The two lateral expression strands of *Acr*-*Hox3* resemble the *Nvi*-*Hox3* and *Pdu-Hox3* expression strands [17]. *Acr*-*Hox4* transcription products appear along the ventral and lateral hyposphere of the polyplacophoran, similar to the ventral and lateral expression of *Nvi*-*Hox4* and *Pdu-Hox4* in the developing second and third parapodial segments and the site of *Acr-Hox5* expression is comparable to that of *Nvi-Hox5* and *Pdu-Hox5* expression in the developing second and third parapodial segment [17]. *Acr*-*Hox7* transcripts are distributed posteriorly in a dorso-ventral ring-like pattern; *Nvi*-*Hox7* and *Pdu-Hox7* are also expressed posteriorly, namely in the developing third parapodial and pygidial segment [17]. *Acr*-*Post2* expression is restricted to the posterior pole, similar to the pygidial expression of *Nvi*-*Post2* and *Pdu-Post2* [17]. In addition to the expression data in *N. virens* and *P. dumerilii,* a relatively similar AP expression pattern is also known from other early annelid developmental stages (e.g., *Chaetopterus* [16], *Helobdella robusta and H. triserialis* [46], *Capitella* sp. [51], *Platynereis* [53]).

Overall, the relative spatial antero-posterior Hox gene expression pattern in early developmental stages of *Nereis virens* and *Platynereis dumerilii*, as well as other annelids, is comparable to that of the polyplacophoran *Acanthochitona crinita*. In particular, the spatial antero-posterior Hox gene expression pattern in early polyplacophoran trochophores is also found in the ecto-, endo- and mesodermal cell layers and is thus not restricted to specific organs. Annelid Hox gene expression in the nervous system appears for the first time in meta-trochophore larvae [16, 17, 46, 51].

## Conclusion

The relatively similar co-linear Hox gene expression signature in Polyplacophora, Annelida and other bilaterians demonstrates that the ancestral role of Hox genes in Mollusca was also in patterning of the antero-posterior body axis. After the aculiferan-conchiferan split, the Hox genes were secondarily recruited into novel functions in gastropods and cephalopods (either individually or at the base of Conchifera), but data on bivalves, scaphopods and monoplacophorans are needed to further substantiate this assumption. Overall, the plasticity of Hox gene functions in mollusks may well explain the high morphological variability exhibited among the various molluscan sub-lineages and may thus have been an important driving force for the evolutionary success of gastropods and cephalopods.

## Methods

### Rearing and larval development of *Acanthochitona crinita*

Adult *A. crinita* were collected at the Biological Station Roscoff, Brittany, France. Spawning was induced by alternating hot (30–40 °C) and cold (3–6 °C) shocks as well as sunlight exposure. After spawning, one to two drops of concentrated sperm solution was used to fertilize the eggs in 150–200 ml seawater for 30 min at 21–23 °C. After spawning and fertilization, developmental stages were reared in filtered seawater. First cleavage stages appeared 3–5 h after fertilization. First trochophore larvae hatched nine to 10 h after fertilization and post-metamorphic animals appeared three to 4 days after fertilization.

### Fixation and RNA extraction

Developmental stages were fixed in 4 % paraformaldehyde in MOPS buffer (0.5 M Mops, 10 mM MgSO4, 5 mM EGTA, 2.5 M NaCl) for 45 min at room temperature, stepped into 100 % methanol and stored in 100 % methanol at −20 °C. To extract RNA with the RNeasy mini Kit (#74104,Qiagen), a pool of representative developmental stages was used. RNA was sequenced by Illumina technology and reads were assembled into contigs by Trinity [58].

### Orthology assignment and phylogenetic analysis of Hox genes

The assembled sequences from the *Acanthochitona crinita* transcriptome were used in local similarity searches using the program tblastn [59] against known and well-curated Hox sequences retrieved from GenBank non-redundant protein database. The top 10 BLAST hits of each similarity search were individually and manually analyzed. The Hox domain-containing sequences were assigned according to the presence of diagnostic residues/motif in the homeodomain as well as in the flanking regions and through Bayesian molecular phylogenetic analysis (Fig. 1). The program Jalview 2 [60] was used to illustrate the multiple sequence alignment of the Hox genes. All sequences of species used in the phylogenetic analysis and corresponding accession numbers are listed in Table 1. The Bayesian analyses were carried out with MrBayes v3.2.2 software [61] with Jones-Taylor-Thornton model of amino-acid substitution [62], gamma-distributed rates, 25,000,000 generations and sampling frequency of 1000.

**Table 1:** Species, genes and GenBank accession numbers used in the phylogenetic analysis

*Species name/Abbreviation*	Phylum	*Gene Name*	Accession Number
*Acanthochitona crinita*/*Acr*	Mollusca	*Acr_Hox1*	KR190463
		*Acr_Hox2*	KR190464
		*Acr_Hox3*	KR190465
		*Acr_Hox4*	KR190466
		*Acr_Hox5*	KR190467
		*Acr_Hox7*	KR190468
		*Acr_Post2*	KR190469
*Branchiostoma floridae*/*Bfl*	Chordata	*Bfl_Hox1*	BAA78620
		*Bfl_Hox2*	BAA78621
		*Bfl_Hox4*	BAA78622
		*Bfl_Hox5*	ABX39489
		*Bfl_Hox7*	ABX39491
*Bugula turrita*/*Btu*	Bryozoa (Ectoprocta)	*Btu_Hox2*	AAS77225
		*Btu_Hox3*	AAS77226
		*Btu_Hox4a*	AAS77227
		*Btu_Hox4b*	AAS77228
		*Btu_Post2*	AAS77230
*Caenorhabditis elegans*/*Cel*	Nematoda	*Cel_Hox1*	CAA34929
*Ciona intestinalis*/*Cin*	Chordata	*Cin_Hox1*	NP_001122333
		*Cin_Hox2*	CAD59668
		*Cin_Hox4*	NP_001027781
		*Cin_Hox5*	NP_001027665
*Drosophila melanogaster*/*Dme*	Arthropoda	*Dme_Hox1*	CAB57787
		*Dme_Hox2*	CAA45271
		*Dme_Hox3*	P09089
		*Dme_Hox4*	P07548
		*Dme_Hox5*	NP_524248
		*Dme_Hox7*	CAA27417
		*Dme_Dll*	NP_726486
*Euprymna scolopes*/*Esc*	Mollusca	*Esc_Hox1*	AAL25804
		*Esc_Hox3*	AAR16188
		*Esc_Hox5*	AAR16189
		*Esc_Hox7*	AAL25809
		*Esc_Post2*	AAL25812
*Flaccisagitta enflata*/*Fen*	Chaetognatha	*Fen_Hox1*	ABS18809
		*Fen_Hox3*	ABS18810
		*Fen_Hox4*	ABS18811
		*Fen_Hox5*	ABS18812
*Gibbula varia*/*Gva*	Mollusca	*Gva_Hox1*	ACX84671
		*Gva_Hox2*	ADJ18233
		*Gva_Hox3*	ADJ18232
		*Gva_Hox4*	ACX84672
		*Gva_Hox5*	ADJ18234
		*Gva_Hox7*	ADJ18235
		*Gva_Post2*	ACX84674
*Haliotis asinina*/*Has*	Mollusca	*Has_Hox3*	AAK17185
		*Has_Hox4*	AAK11240
		*Has_Hox5*	AAF78248
*Lingula anatina*/*Lan*	Brachiopoda	*Lan_Hox1*	AAD45587
		*Lan_Hox3*	AAD45588
		*Lan_Hox5*	AAD45589
		*Lan_Hox7*	AAD45590
		*Lan_Post2*	AAD45595
*Lithobius atkinsoni*/*Lat*	Arthropoda	*Lat_Hox1*	AAL36907
		*Lat_Hox2*	AAL36908
		*Lat_Hox3*	AAL36906
		*Lat_Hox4*	AAL36902
		*Lat_Hox5*	AAL36909
		*Lat_Hox7*	AAL36901
*Lineus sanguineus*/*Lsa*	Nemertea	*Lsa_Hox1*	CAA76295
		*Lsa_Hox3*	CAA76296
*Mus musculus*/*Mmu*	Chordata	*Mmu_Hox1*	NP_034579
		*Mmu_Hox2*	NP_034581
		*Mmu_Hox3*	NP_034582
		*Mmu_Hox4*	NP_032291
		*Mmu_Hox5*	NP_034583
		*Mmu_Hox7*	NP_034585
*Nymphon gracile*/*Ngr*	Arthropoda	*Ngr_Hox1*	ABD46723
		*Ngr_Hox2*	ABD46725
		*Ngr_Hox5*	ABD46729
		*Ngr_Hox7*	ABD46732
*Nereis virens*/*Nvi*	Annelida	*Nvi_Hox1*	AAD46166
		*Nvi_Hox2*	AAD46167
		*Nvi_Hox4*	AAD46169
		*Nvi_Hox5*	AAD46170
		*Nvi_Hox7*	ABD04657
		*Nvi_Post2*	AAD46176
*Platynereis dumerilii*/*Pdu*	Annelida	*Pdu_Hox3*	ABD04656
		*Pdu_Hox4*	ABD04658
		*Pdu_Hox5*	ABD04660
		*Pdu_Post2*	ABD04651
		*Pdu_Dlx1*	CAJ38799
*Sacculina carcini*/*Sca*	Arthropoda	*Sca_Hox1*	ABB46347
		*Sca_Hox2*	AAD00340
		*Sca_Hox4*	AAD00345
		*Sca_Hox5*	AAM50457
*Saccoglossus kowalevskii*/*Sko*	Hemichordata	*Sko_Hox1*	AAP79296
		*Sko_Hox2*	ABK00018
		*Sko_Hox3*	AAP79286
		*Sko_Hox4*	AAP79297
		*Sko_Hox5*	ABK00019
		*Sko_Hox7*	AAP79287
*Symsagittifera roscoffensis*/*Sro*	Acoelomorpha	*Sro_Hox1*	AAN11404
*Tribolium castaneum*/*Tca*	Arthropoda	*Tca_Hox1*	NP_001107762
		*Tca_Hox3*	AAK16424
		*Tca_Hox4*	AAK16423
		*Tca_Hox5*	AAK16422

### Primer design and probe synthesis

*Acr-Hox* gene-specific primer design was performed with the sequence assembling software Geneious 6.1.6 (Biomatters Limited) and primers were purchased from Life Technologies Company (Thermo Fischer Scientific). First strand cDNA was synthesized by reverse transcription of RNA pooled from representative developmental stages covering the entire larval and early post-metamorphic development (cDNA synthesis kit, #04379012001, Roche Diagnostics). Hox gene sequences were amplified with the gene-specific primers via standard PCR. PCR products were cloned by insertion into pGEM-T easy vectors (#A1360, Promega) and plasmid minipreps were purified with Qia-miniprep kit (#27106, Qiagen). Antisense and sense probes from linearized plasmid sequences were synthesized with a DIG-labeling kit (#11277073910, Roche Diagnostics).

### Whole mount in situ hybridization

*Acanthochitona crinita* trochophore larvae were decalcified in ME (90 % MeOH and 50 mM EGTA) for 10 min and in PPE (4 % PFA, 1 × PBS, 50 mM EGTA) for 45 min and subsequently washed in PBT (1 × PBS, 0.1 % Tween 20). Larvae were incubated in a 60 μg/ml proteinase-K solution at 37 °C for 10 min and then washed in 0.2 % glycine in PBT. To reduce charged probe binding, larvae were put in 1 % triethanolamine and 0.5 % acetic anhydride solution, washed in PBT and post-fixed for 45 min in 4 % PFA. Afterwards, larvae were stepped into 100 % hybridization buffer and pre-incubated at 60 °C in a water bath overnight. Larvae were hybridized with antisense probes which are complementary to the transcript sequence or with sense probes as negative controls (0.25–0.7 ng/μl probe concentration) at 60 °C for 48 h. After hybridization, larvae were washed several times in 4 × Wash (25 % formamide, 4 × SSC, 0.1 % Tween 20), 2 × Wash (with 2 × SSC) and 1 × Wash (with 1 × SSC). Afterwards, specimens were stepped into and washed several times in 1 × SSC, then several times in MAB (100 mM maleic acid, 150 mM NaCl, 0.02 % Tween 20). Larvae were washed in 2 % blocking solution (#11096176001, Roche) in MAB for 2–3 h and incubated in 1:5000 DIG antibody solution (#11093274910, Roche) at 4 °C overnight, then washed several times in PBT, incubated twice in an AP-buffer (1× alkaline phosphatase buffer, 0.1 Tween 20) and twice in an AP-buffer with MgCl_2_. RNA transcripts were visualized with a color reaction buffer (1× AP-buffer, 7.5 % polyvinylalcohol, 2 % NBT/BCIP (#11681451001, Roche)); the reaction was stopped with PBT washing steps and then larvae were post-fixed in 4 % PFA at 4 °C overnight. Larvae were cleared in 1:1 benzylalcohol:benzylbenzoate mixture and mounted on glass slides.

Stained larvae were documented with a Nikon Eclipse E800 microscope and a Nikon Fi2-U3 camera. Additionally, larvae were scanned with a Leica DMI6000 CFS confocal laser scanning microscope equipped with a Leica TCS SP5 II scanning system. Specific NBT/BCIP stainings were detected with 633 nm laser wavelength reflection scanning [63]. Confocal stacks were edited using IMARIS 7.3.1 (Bitplane AG) and figure plates were assembled using Coral Graphic Suite X3 (Corel Corporation).
